# Practical Guide to Integrating Geriatric Assessment in Gastrointestinal Oncology in a Resource-Limited Setting

**DOI:** 10.3390/jcm14238448

**Published:** 2025-11-28

**Authors:** Radu Vidra, Paula-Viorica Alexander, Gábor Liposits

**Affiliations:** 1 MEDFUTURE Biomedical Research Institute, Department of Personalized Medicine and Rare Diseases, Iuliu Hatieganu University of Medicine and Pharmacy, 400347 Cluj-Napoca, Romania; radu.vidra@irgh.ro; 2 Department of Medical Oncology, Regional Institute of Gastroenterology and Hepatology “Prof. Dr. Octavian Fodor”, 400394 Cluj-Napoca, Romania; 3 UBBMed, The International Institute for the Advanced Studies of Psychotherapy and Applied Mental Health, Babes-Bolyai University, 400347 Cluj-Napoca, Romania; 4 Oncology Institute “Prof. Dr. Ion Chiricuta”, 400015 Cluj-Napoca, Romania; 5 Department of Oncology, San Maurizio Central Hospital in Bolzano, South Tyrolean Health Service, 39100 Bolzano, BZ, Italy

**Keywords:** gastrointestinal cancer, geriatric assessment, frailty screening, G8, VES-13, personalized oncology, resource-limited settings

## Abstract

**Background/Objective:** The global burden of gastrointestinal (GI) cancers is rising sharply among the elderly, with a projected doubling of incidence and mortality by 2050. Given the heterogeneity of aging and the limitations of traditional performance scales such as ECOG or KPS, integrating geriatric assessment (GA) into oncology has become essential for tailoring safe and effective treatment strategies in this population. This paper provides a practical framework for implementing geriatric assessment and management (GAM) in GI oncology, particularly in resource-limited settings, and highlights validated screening instruments suitable for clinical integration. **Methods:** A systematic search was performed across PubMed, Scopus, and Web of Science databases for publications between January 2019 and August 2025 using combinations of the keywords geriatric assessment, gastrointestinal cancer, frailty screening, elderly, oncology, and comprehensive geriatric assessment. International and regional clinical practice guidelines from ASCO, ESMO, and SIOG were reviewed in detail. Articles were included when they addressed validated screening tools, oncology focused strategies, or clinical outcomes associated with GA-based interventions. Studies focusing exclusively on non-oncologic geriatric populations were excluded. Relevant data were extracted regarding study design, population, tool validation, predictive performance, and feasibility. **Results:** GA improves prediction of treatment-related toxicity, supports individualized treatment planning, and enhances quality of life and functional outcomes. Two-step screening approaches, initial frailty screening followed by comprehensive geriatric assessment for those with positive results, were found most effective. Practical GA models and telehealth-based applications were identified as feasible even in low- and middle-income contexts. **Conclusions:** Integrating GA into GI oncology fosters patient-centered, evidence-based care that optimizes treatment tolerance, reduces complications, and aligns therapeutic goals with patient values. Institutional commitment, interdisciplinary collaboration, and targeted training are pivotal for establishing GA as a standard of care across diverse healthcare settings.

## 1. Introduction

Gastrointestinal (GI) cancers represent an important and growing global health burden, particularly among the elderly [1,2,3]. According to projections from the Global Cancer Observatory, the number of GI cancer cases in adults aged 65 and over is expected to rise from 2.96 million in 2022 to 6.37 million by 2050, with cancer-related deaths increasing from 2.16 million to 4.81 million over the same period [4].

This course emphasizes the impact of aging combined with repeated exposure to modifiable risk factors, such as smoking, obesity, and alcohol consumption. The growing burden highlights the need for early detection strategies and integration of geriatric tools in routine oncology care to better address the complexity and vulnerability of elderly with cancer [5].

Elderly represent a particularly vulnerable population in oncology, often presenting with multiple comorbidities, altered pharmacokinetics, and increased susceptibility to treatment-related toxicity [6]. Therefore, clinicians face significant complexity when it comes to therapeutic decision-making in elderly [7].

Age-related heterogeneity in functional status, comorbidities, polypharmacy, and geriatric syndromes contribute to considerable differences in treatment approaches and clinical outcomes. As a result, there is a risk of both undertreatment, potentially compromising effectiveness, while overtreatment may increase the risk of adverse events [8]. Such imbalances influence the risk of impaired quality of life and compromise overall survival [7].

Despite the vulnerability of older cancer patients, current healthcare models and clinical practice guidelines remain predominantly disease-centered, and patient-related factors remain left out of consideration when management of the patient is being planned [6,9].

Recent evidence published between 2022 and 2025 emphasizes the growing clinical relevance of integrating geriatric assessment into gastrointestinal oncology. The 2024 ESMO/SIOG position paper underlined that comprehensive geriatric assessment (CGA) not only improves treatment personalization but also reduces therapy related toxicity and enhances functional independence. Furthermore, ASCO’s 2023 guideline update highlights the feasibility of abbreviated screening tools such as the G8 or VES-13 for daily practice, even in outpatient and resource limited settings. Recent studies have also explored the role of digital and telehealth-based GA interventions, which have demonstrated comparable reliability to in-person assessments and offer a scalable solution to extend geriatric oncology care.

To address these challenges, integrating geriatric oncology principles into all levels of clinical care and training is essential. Geriatric assessment and management (GAM) represents a holistic approach to the patients, systematically evaluating domains not covered by past medical history or routine physical examination, including functional status, comorbidities, medications, nutritional status, social functionality, social support, psychological status, cognitive function including depression, and geriatric syndromes [8,10]. Patients may experience improvement or worsening of symptoms during cancer treatment, and therefore periodic geriatric reassessment and co-management through interventions are important. Periodic geriatric reassessment at disease progression or when alteration in functional status occurs may assist decision-making, both including modification of therapeutics (increasing or decreasing dose or delaying treatment) and initiating supportive or even palliative care interventions [8].

Traditional performance measures like the Eastern Cooperative Oncology Group (ECOG) and Karnofsky Performance Status (KPS) scores often fail to detect the complexity of aging and functional decline [10]. Given time constraints in routine oncology practice, validated, brief, easy-to-use screening tools such as the Geriatric 8 (G8) and the Vulnerable Elders Survey-13 (VES-13) offer a practical set of tools to identify patients who may benefit from a full geriatric assessment and management (GAM) [11,12].

Although the approach consisting of initial frailty screening followed by comprehensive geriatric assessment is already endorsed by major international societies, the present manuscript provides an added contribution by situating these recommendations within the specific clinical context of gastrointestinal oncology and by emphasizing their applicability in resource-constrained environments. The originality of this work does not derive from proposing a novel assessment algorithm, but rather from synthesizing recently accumulated evidence (2022–2025) and translating it into a practical, clinically actionable framework adapted to the realities of oncology departments that lack systematic geriatric support. By integrating updated validation data on widely used screening instruments, clarifying their relative strengths and limitations, and discussing feasibility considerations drawn from routine practice, this perspective addresses a persistent gap between guideline-level recommendations and their actual implementation in day-to-day patient care. In this sense, the manuscript offers an academically grounded yet operational interpretation of existing principles, thereby supporting more consistent and equitable incorporation of geriatric assessment into GI oncology.

## 2. Material and Methods

This article was developed as a narrative, expert-informed perspective grounded in a targeted review of the literature on geriatric assessment and management in gastrointestinal oncology. To gather the evidence summarized in this manuscript, we performed a structured search of PubMed, Scopus, and Web of Science, covering publications from January 2019 to August 2025. The search used predefined keyword combinations relevant to the topic, including geriatric assessment, frailty screening, gastrointestinal cancer, elderly, G8, VES-13, comprehensive geriatric assessment, and oncogeriatrics. The search strategy was adapted to each database and was complemented by manual screening of reference lists from key articles and guideline documents.

Studies were included if they addressed validated geriatric screening instruments, practical geriatric assessment models, clinical outcomes informed by geriatric evaluation, or implementation strategies relevant to oncology. We excluded publications focusing exclusively on non-oncologic geriatric populations or those not applicable to gastrointestinal cancers. Data extraction focused on study type, population, assessment tools, predictive or clinical utility, and feasibility in routine clinical care, especially in resource-limited environments. Because this manuscript provides a pragmatic synthesis rather than a systematic review or meta-analysis, no quantitative pooling, scoring of methodological quality, or formal risk-of-bias assessment was performed.

Reference management and removal of duplicates were performed using EndNote 21 (Clarivate Analytics, Philadelphia, PA, USA, 2024 edition). No additional software was required for data analysis, and no preregistration was applicable, as this work is not based on a prospectively defined protocol or original data collection.

## 3. Results and Discussion

The implementation of a geriatric assessment and management (GAM) is recommended in the care of elderly individuals with cancer by the International Society of Geriatric Oncology (SIOG) and the American Society of Clinical Oncology (ASCO) [13]. Performed by a geriatrician or a trained healthcare professional, this multidimensional evaluation assesses functioning (physical, cognitive, social, and emotional), comorbidities, polypharmacy, depression, fatigue, nutrition, and geriatric syndromes. Designed to gain a holistic understanding of the patient’s overall health and to inform a personalized, patient-centered care plan that addresses both oncologic and non-oncological needs [14], these screening tools can effectively distinguish between fit elderly with cancer who are suitable for standard oncologic therapies and vulnerable patients, who require a comprehensive geriatric assessment to guide the development of an individualized and appropriate treatment strategy [15].

Choosing the screening tool (or combination of tools) (Table 1) should take into account local facilities, available resources, and staff skills and experience, and should be adapted to what is feasible in the clinical setting or to the specific clinical or research needs [16]. In clinical practice, the Geriatric 8 (G8) and the Vulnerable Elderly Survey-13 (VES-13) are the most commonly used and well-validated geriatric screening tools [16,17].

### 3.1. G8 (Geriatric 8) and Modified G8

Derived from the Mini Nutritional Assessment (MNA), the G8 is a validated screening tool specifically developed for elderly individuals with cancer to identify those who may benefit from a GAM [18,19]. It is noted for its high sensitivity but low specificity [17,20]. It evaluates eight domains: food intake, weight loss over the past 3 months, mobility, neuropsychological status, body mass index, number of daily medications, self-perceived health status, and age [18]. A score of ≤14 suggests the need for further geriatric evaluation. Recently, a self-administered version of the G8, known as the Self-G8, has been developed, maintaining the same components and demonstrating comparable performance in detecting frailty. The G8 modified index demonstrated very good diagnostic performance in identifying vulnerable older cancer patients and has been externally validated as a reliable screening tool in geriatric oncology [15,20]. The G8 screening tool has proven effective in predicting mortality, survival, functional decline, chemotherapy-related toxicity, and treatment trajectory across different tumor types and clinical settings [21]. While it has demonstrated prognostic value in solid tumors, its predictive performance remains limited in hematological malignancies [22,23,24].

### 3.2. VES-13 (Vulnerable Elders Survey-13)

Also a strongly validated and widely accepted instrument, the Vulnerable Elders Survey-13 (VES-13) was designed to identify frailty in the elderly. It serves as a valuable predictor of functional decline, institutionalization, and mortality. VES-13 is both efficient and accessible, requiring less than 5 min to complete, and can be conducted either face-to-face or by telephone, directly with the patient or through a knowledgeable proxy [25]. The four essential domains evaluated here are age, limitations in daily activities, self-rated overall health, and physical functioning [11,26]. The tool yields a score from 0 to 10, using the answers received from 13 straightforward questions. Results with a score of ≥3 indicate significant vulnerability and in turn warrant further geriatric investigation, meaning the threshold of three or higher indicates frailty. In clinical practice, the VES-13 supports early recognition of vulnerable individuals, enabling timely interventions that may preserve autonomy and quality of life in an aging population [25]. It was significantly associated with severe chemotherapy-related toxicity in patients with diverse malignancies [27,28]. and predicted overall survival in elderly with digestive cancers receiving chemotherapy [29]. The combined use of VES-13 and G8 has shown promise as a sensitive approach to identifying older cancer patients at risk of vulnerability. When at least one of the tools indicated impairment, the screening achieved a sensitivity of 86.6% and a specificity of 53.2% in detecting unfit patients for standard treatment following comprehensive geriatric assessment [30,31].

### 3.3. Timed up and Go (TUG)

To estimate mobility and the risk of falls, a useful method of assessing is the Timed Up and Go (TUG) test, a standardized and efficient functional evaluation that is particular to the elderly. It consists of the individual having to stand up from a chair, walk 3 m, turn, return to the chair, and sit down, where the indicator of functional performance is the total time it takes the individual to finish. Clocking 12 s will generally be associated with reduced mobility and an increased risk of falling. In recent years, TUG tests can independently be performed at home through the development of mobile applications, which offers a convenient and accessible method of early functional decline detection and continuous monitoring. In elderly undergoing surgery for solid tumors, the Timed Up and Go (TUG) test effectively predicted postoperative morbidity, performing comparably to a full geriatric assessment in identifying high-risk individuals [32].

### 3.4. Mini-Cog

The Mini-Cog is a brief cognitive screening tool that combines a three-item recall task with a simplified clock drawing test. It is widely used to detect cognitive impairment in elderly people with cancer, with reported prevalence ranging from 2.6% to 52% in this population. One of its main advantages is its brevity, requiring only 3–5 min to complete, which makes it practical for routine clinical use. The Mini-Cog demonstrates minimal educational bias and maintains good diagnostic accuracy across diverse patient groups. It also has predictive value; a score of ≤2 has been associated with a significantly increased risk of postoperative delirium in older surgical cancer patients. However, the Mini-Cog assesses only basic cognitive domains and may not detect subtle deficits or mild cognitive impairment. Its performance can also be influenced by cultural or linguistic factors, limiting its applicability in some populations. Importantly, while the Mini-Cog is a useful screening instrument, it does not replace comprehensive neuropsychological testing for definitive diagnosis. Overall, the Mini-Cog is a valuable tool in geriatric oncology for identifying patients at risk of cognitive decline and guiding individualized treatment planning, but it should be integrated into a broader geriatric assessment framework [6].

### 3.5. Cancer and Aging Research Group (CARG) Toxicity Score

To estimate the risk of severe chemotherapy-related toxicity in elderly individuals with cancer, a tool called The Cancer and Aging Research Group (CARG) toxicity score was developed [33,34,35]. It uses a combination of and geriatric evaluation parameters, including age, sex, cancer type, chemotherapy regimen, creatinine clearance, hemoglobin level, mobility, history of falls, level of social activity, and hearing ability [36]. Categorizing patients into low- (0–5), intermediate- (6–9), and high-risk (10–19) groups, the total score ranges from 0 to 19 [37]. This tool has been validated across multiple studies and has shown greater predictive accuracy than traditional assessment methods such as the ECOG performance status and the age-adjusted Charlson Comorbidity Index [33,38].

Older patients have a higher incidence of severe chemotherapy-related toxicity compared to younger individuals. Accurate risk estimation is essential for optimizing therapeutic decisions. In the absence of reliable predictors, clinicians may adopt a cautious approach, increasing the risk of undertreatment [39,40].

### 3.6. Practical Geriatric Assessment—ASCO

There are several advantages; e.g., it does not require geriatric expertise, it is a questionnaire a patient can complete by themselves or with assistance, and it can be applied in resource-limited settings. It can be a standard in daily practice as well as in research, allowing better comparison of frailty in different cohorts and interpretation of results [41].

### 3.7. Treatment Adjustments

The attending physician may, at their discretion, suggest any modifications that should be implemented and will also determine the appropriate course of action based on clinical status, disease progression, treatment response, comorbidities, and patient preferences. Shared decision-making is one of the most important factors, considering the patient’s values, goals, and preferences (Table 2).

Prospective evidence suggests that implementing planned dose reductions at treatment initiation for older, vulnerable patients can maintain antitumor efficacy while substantially improving treatment tolerability and patient-reported outcomes. In the NORDIC9 trial, reduced-dose combination chemotherapy demonstrated progression-free survival comparable to full-dose monotherapy, and a more favorable toxicity profile [42]. Follow-up quality-of-life analyses from the same study further corroborated these findings, indicating stable or improved global quality of life, functional status, and symptom burden, with fewer treatment-related adverse events [43]. Consistent results were observed in the phase III GO2 trial, in which lower-intensity oxaliplatin–capecitabine regimens were non-inferior for cancer control and conferred meaningful preservation of quality of life in older, frail patients with advanced gastroesophageal tumors. Taken together, these data provide robust support for geriatric-adapted upfront dose strategies as a rational and patient-centered approach in gastrointestinal oncology.

## 4. Conclusions

Integrating geriatric assessment into the management of older adults with gastrointestinal cancers provides safe, effective, and patient-centered oncology care. Given the complexity and heterogeneity of the aging process, exclusive reliance on chronological age or traditional performance status scores is insufficient.

This article contributes an innovative and pragmatic perspective by outlining a structured screening and assessment model that can be implemented even in resource-limited oncology services. The integration of simplified screening tools such as the G8, VES-13, and TUG, followed by comprehensive geriatric assessment (CGA) in positive cases, enables a scalable and reproducible framework for personalized oncology.

### 4.1. Stepwise Implementation in Resource-Limited GI Oncology Settings

In resource-constrained environments, a pragmatic, low-cost workflow is essential. We propose a simple model that can be implemented even in the absence of dedicated geriatric staff.

1. Who and when to screen

All patients aged ≥ 70 years (or ≥65 years with at least two relevant comorbidities) should be screened at the first oncology visit and at major decision points in the treatment trajectory (initiation of systemic therapy, preoperative assessment, or at disease progression).

2. How to screen with limited personnel
  - The G8 (or VES-13 in centers already familiar with it) can be completed by the patient or caregiver in the waiting room or during pre-visit telephone contact, with a nurse or a junior doctor checking for completeness.
  - The TUG and Mini-Cog are performed only when the G8/VES-13 result is abnormal and can be administered by a nurse, physiotherapist, or trained medical student in under 5 min.
  - All proposed tools are freely available, paper-based, and do not require proprietary software or equipment.
3. Follow-up algorithm for positive screens
  - Normal screening: Proceed with standard oncologic assessment and treatment.
  - Mildly abnormal G8/VES-13 with preserved function: Management remains within the oncology team, focusing on targeted measures such as planned dose reductions, nutritional counseling, and a basic review of polypharmacy.
  - Markedly abnormal scores and/or impaired TUG/Mini-Cog: Refer for a comprehensive geriatric assessment where available, or, at minimum, implement a structured bundle of interventions (detailed medication review, referral to physiotherapy/occupational therapy, social work involvement, and early integration of supportive/palliative care).

This stepwise approach provides a concrete, reproducible framework that can be adapted to different resource levels while maintaining feasibility in busy GI oncology clinics.

Comprehensive Geriatric Assessment (CGA) offers a holistic approach, enabling a better understanding of each patient’s physiological reserve and treatment tolerance. The benefits of this strategy are increasingly recognized and include

- Improved prediction of treatment toxicity and functional decline;
- The development of individualized treatment plans, including appropriate dose modifications, therapy selection, and de-intensification strategies where appropriate;
- A reduction in complications and unplanned hospitalizations;
- Higher rates of treatment completion and maintenance of quality of life;
- Better communication and targeted planning for supportive and end-of-life care.

To improve the outcomes and also align therapeutic decisions with patient values and life goals, the geriatric tools should be introduced as the norm of standard oncology. Thus, systematic implementation of screening and CGA in GI oncology should therefore become a routine standard of care, conferred by interprofessional collaboration, dedicated training, and institutional commitment.

Even in resource-limited settings, the use of brief screening tools followed by targeted, high-impact interventions can significantly improve treatment tolerance and support functional preservation. The evidence reviewed highlights that simplified, scalable GA workflows, supported by task-shifting and minimal essential follow-up pathways, are both feasible and beneficial.

### 4.2. Pragmatic Recommendation Regarding Choice of Screening Tools

For everyday practice in gastrointestinal oncology, particularly in resource-limited settings, we suggest using the G8 as the default initial screening instrument for elderly with cancer. Where feasible, this may be complemented by a brief mobility test such as the Timed Up and Go (TUG) and a short cognitive screen such as the Mini-Cog, in order to capture domains most strongly associated with treatment tolerance. In centers that are already familiar with the VES-13, this tool represents a valid alternative to, or complement for, the G8. For patients who are candidates for systemic anticancer therapy, we recommend, when available, integrating a chemotherapy toxicity risk score such as the CARG tool in addition to frailty screening. Any abnormal result on these instruments should trigger either a comprehensive geriatric assessment or, when this is not possible, at least minimal, targeted geriatric interventions.

## 5. Limitations

This guide is based on published evidence and expert experience, which may limit its generalizability across different healthcare systems. The proposed geriatric assessment approach has not been prospectively validated in resource-limited settings, and the availability of trained personnel, tools, and institutional infrastructure may differ substantially between centers. In addition, some of the referenced geriatric instruments lack extensive validation in populations with advanced gastrointestinal cancers. Further prospective studies are needed to evaluate feasibility and clinical impact in diverse real-world environments.

## Figures and Tables

**Table 1:** Geriatric screening tools.

Geriatric Screening Tools:
● Geriatric 8 (G8)
● Vulnerable Elders Survey (VES-13)
● Timed Up and Go (TUG)
● Mini-Cog
● Flemish version of Triage Risk Screening Tool (fTRST)
● Groningen Frailty Indicator (GFI)
● Fried Frailty Criteria
● Handgrip strength
● Study of Osteoporotic Fracture (SOF)
● Barber Questionnaire
● Identification of Seniors at Risk (ISAR)
● Senior Adult Oncology Program 2 (SAOP2)
● Physical Performance Test (PPT)
● SPICES–Skin integrity; Problems eating; Incontinence; Evidence of falls; Sleep disturbance
● Blessed Orientation Memory Concentration (BOMC)

**Table 2:** Treatment adjustments.

Patients being considered for chemotherapy/targeted therapy:
● Standard regimen at reduced dose
● Less toxic regimen at standard dose
● Less toxic regimen at reduced dose
● No chemotherapy
● Patients being considered for surgery
● Minimal invasive techniques
● Enhanced recovery protocols
● Less extensive surgery
● Delayed surgery
● No surgery
Patients being considered for Radiotherapy
● Radiotherapy instead of surgery/chemotherapy
● Radiotherapy alone instead of concomitant radiochemotherapy
Patients being considered for hormonal therapy
● Different choice of hormonal therapy
● Hormonal therapy instead of surgery/chemotherapy
Non-oncologic interventions:
● Polypharmacy optimization—deprescribing
● Management of comorbidities
● Nutritional support
● Social interventions

## Data Availability

This manuscript does not involve the generation or analysis of new datasets. All data discussed are derived from previously published studies cited in the manuscript.

## Abbreviations

ASCO	American Society of Clinical Oncology
BOMC	Blessed Orientation Memory Concentration
CARG	Cancer and Aging Research Group
CCI	Charlson Comorbidity Index
CGA	Comprehensive Geriatric Assessment
CT	Chemotherapy
ECOG	Eastern Cooperative Oncology Group Performance Status
ERAS	Enhanced Recovery After Surgery
ESMO	European Society for Medical Oncology
GA	Geriatric Assessment
GAM	Geriatric Assessment and Management
GFI	Groningen Frailty Indicator
GI	Gastrointestinal
G8	Geriatric 8 Screening Tool
HT	Hormonal Therapy
ISAR	Identification of Seniors at Risk
KPS	Karnofsky Performance Status
MNA	Mini Nutritional Assessment
PFS	Progression-Free Survival
PPT	Physical Performance Test
RT	Radiotherapy
SAOP2	Senior Adult Oncology Program 2
SOF	Study of Osteoporotic Fractures index
SIOG	International Society of Geriatric Oncology
SPICES	Skin Integrity, Problems Eating, Incontinence, Confusion, Evidence of Falls, Sleep Disturbance
TUG	Timed Up and Go
VES-13	Vulnerable Elders Survey-13
fTRST	Flemish version of the Triage Risk Screening Tool
